# Hypothyroidism and sinus dysfunction associated with lithium-paliperidone combination therapy for bipolar disorder with psychotic symptoms: a case report

**DOI:** 10.3389/fpsyt.2024.1336100

**Published:** 2024-02-07

**Authors:** Wenjian Wei, Yonglan Yang, Haifeng Wang, Yunbin Yang, Jizhou Liu, Jinfeng Li

**Affiliations:** ^1^ Department of Clinical Psychology, The 2nd People’s Hospital of Honghe Hani and Yi Autonomous Prefecture, Jianshui, China; ^2^ Department of psychiatry, The 2nd People’s Hospital of Honghe Hani and Yi Autonomous Prefecture, Jianshui, China; ^3^ Department of Psychiatry, Hospital for Infectious Diseases, Honghe Hani and Yi Autonomous Prefecture, Mengzi, China

**Keywords:** lithium, paliperidone, hypothyroidism, bradycardia, sinus arrest, bipolar disorder

## Abstract

**Background and aim:**

Lithium is considered to be the first-line treatment for bipolar disorder, and paliperidone was approved for the treatment of schizophrenia and acute bipolar manic/mixed episodes. However, both agents have been associated with thyroid dysfunction and cardiovascular adverse effects like subclinical hypothyroidism, bradycardia, and sinus arrest, even at therapeutic doses.

**Case presentation:**

Here, we reported a case of a 17-year-old Han Chinese female who developed symptomatic hypothyroidism, sinus bradycardia, and sinus arrest while being treated with lithium and paliperidone for bipolar disorder with psychotic features including auditory hallucinations. Her workup suggested that these adverse effects might be related to the combined lithium and paliperidone treatment, although other causes could not be ruled out. After discontinuing both medications, her thyroid function and heart rhythm normalized over 20 days.

**Conclusion:**

To our knowledge, hypothyroidism, sinus bradycardia, and sinus arrest associated with the combined use of lithium and paliperidone had not been reported previously. Further research is warranted to elucidate the potential risks and benefits of this combination therapy for bipolar disorder with psychotic symptoms.

## Introduction

Lithium, a well-established mood stabilizer, has long been used for the treatment of bipolar disorder. It is known to affect thyroid function, with hypothyroidism being a common adverse effect (1). Lithium has also been associated with cardiotoxicity, usually at serum levels above the therapeutic range (>1.5 mmol/L) (1). However, a previous study reported that lithium can induce reversible bradycardia even at typical therapeutic doses (2). Paliperidone, a second-generation antipsychotic (SGA), is the active metabolite of risperidone. It was approved for a broad spectrum of psychiatric conditions, with extrapyramidal symptoms, hyperprolactinemia, and weight gain known to be common adverse effects (3). However, paliperidone has not been approved for children under the age of 18, except for the treatment of schizophrenia in children aged 12 to 17. The patient’s legal representative has given written informed consent for off-label treatment with paliperidone. Paliperidone has rarely been associated with thyroid dysfunction or bradycardia, although the latter has been reported with its parent compound risperidone (4). The mechanism underlying medication-induced bradycardia remains unclear. Here, we present a case of an adolescent female with bipolar disorder who developed subclinical hypothyroidism and sinus dysfunction after the initiation of combination treatment with lithium and paliperidone. She had normal thyroid function and heart rate prior to starting these medications, and her thyroid hormones and heart rate normalized after the medications were discontinued. This case highlights the potential for clinically significant bradycardia even with the usual treatment doses of these agents.

## Case presentation

A 17-year-old Han Chinese female presented to the psychiatric outpatient clinic with a one-week history of dizziness and feeling sluggish. These symptoms were impairing her ability to attend school regularly. Her psychiatric history was significant for a recent hospitalization three months prior for bipolar disorder with psychotic features (auditory hallucinations). The patient met the *Diagnostic and Statistical Manual of Mental Disorders, Fifth Edition* criteria for bipolar I disorder based on the documented presentation of manic episode as well as prior major depressive episodes over the preceding three months. During admission, she was started on lithium carbonate 500 mg twice daily and paliperidone 9 mg (450 mg Chlorpromazine, equivalent dose) once daily for treatment. Two months later, the paliperidone was down titrated to 6 mg (300 mg Chlorpromazine, equivalent dose) once daily. However, one week before her outpatient visit, she started to experience frequent dizziness, chest tightness, and feeling sluggish. She denied any other personal or family medical conditions, smoking, alcohol use, or illicit drug use. However, it was noted these were based on self-report only, and additional clinical documentation was unavailable to confirm past medical conditions. Prior to the initiation of lithium and paliperidone, she had no history of thyroid disease, bradycardia, or other cardiac issues.

**Figure 1:**
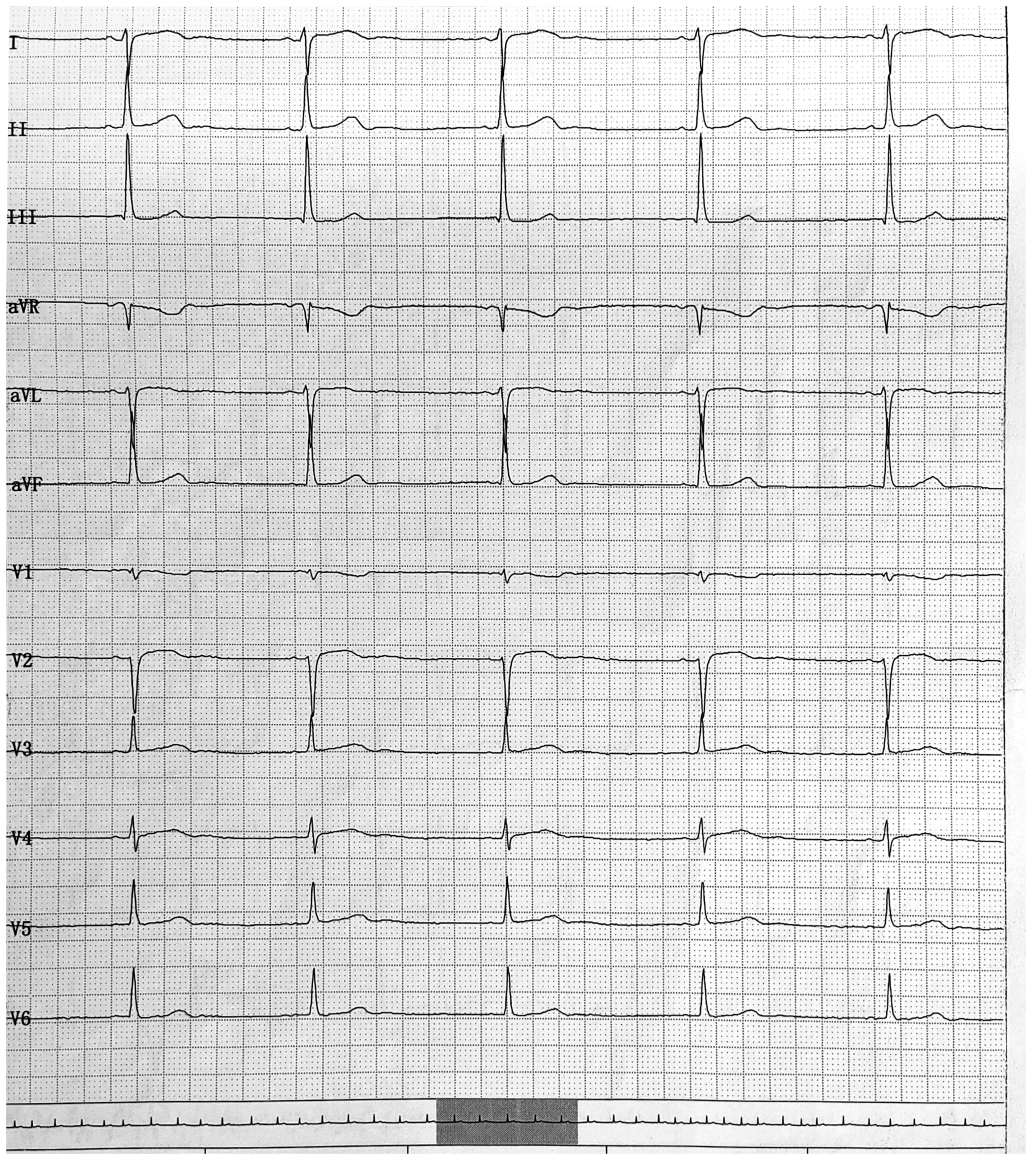
The electrocardiogram shows sinus bradycardia with a heart rate of 41 beats per minute.

**Figure 2:**
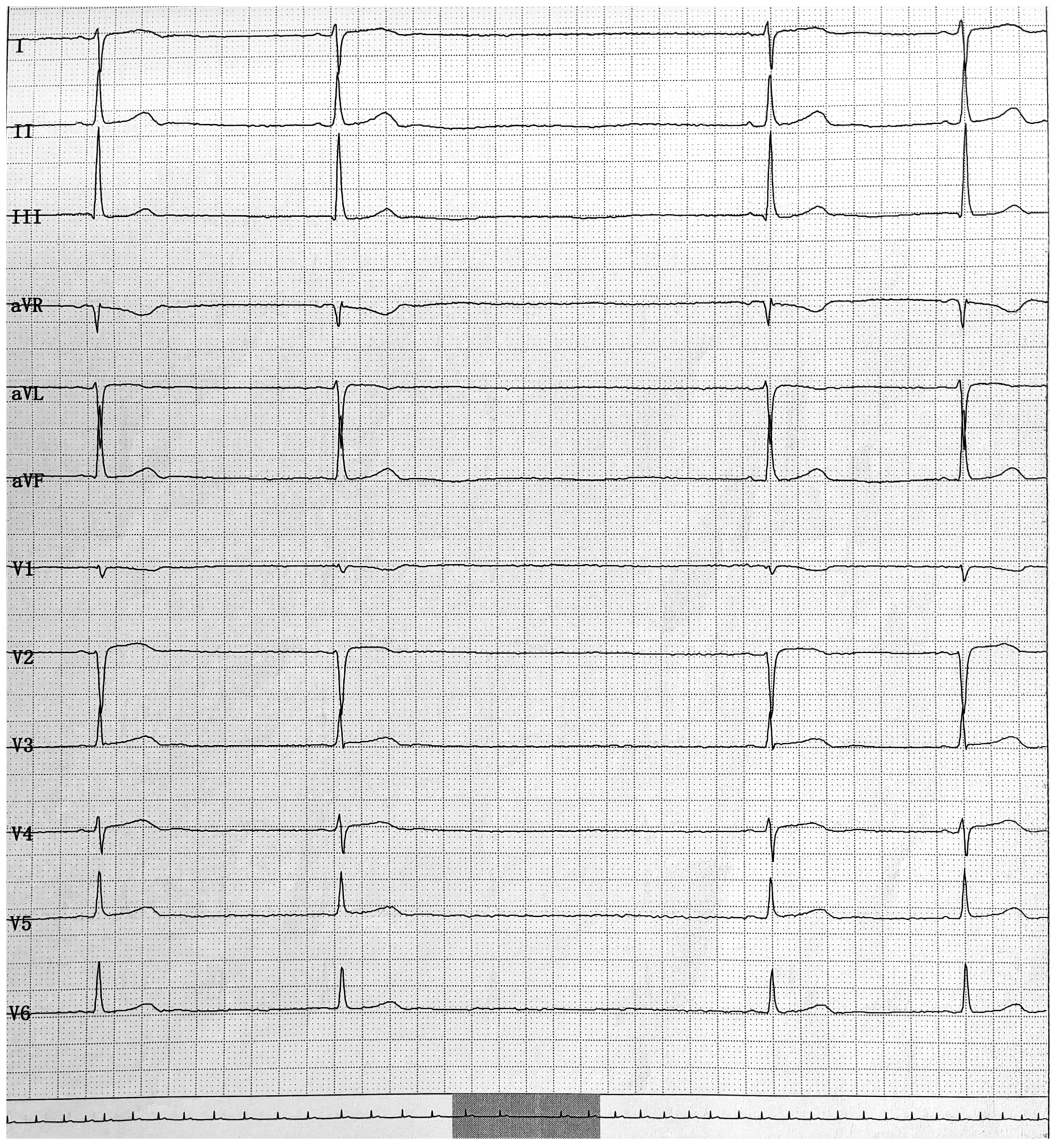
The electrocardiogram shows sinus arrest with an approximate 3.2 second pause.

On physical examination, her heart rate was bradycardic at 41 beats per minute, and her blood pressure was 95/57 mmHg. An electrocardiogram (ECG) revealed sinus bradycardia (**Figure 1**) and sinus arrest (**Figure 2**). Her serum electrolytes, including calcium, potassium, and myocardial enzymes, were within normal limits (**Table 1**). However, thyroid function tests showed an elevated thyroid-stimulating hormone (TSH) of 9.08 mIU/L (reference range 0.51–4.94 mIU/L), decreased total thyroxine (TT4) of 45.05 nmol/L (reference range 58.1–140.6 nmol/L), and decreased free thyroxine (FT4) of 10.94 pmol/L (reference range 11.5–22.7 pmol/L) (**Table 1**). Her thyroid abnormalities were considered subclinical in nature since overt symptomatic manifestations were not charted in the available history. She and her guardian denied any personal or family history of cardiac or thyroid diseases, and she was not taking any other medicines known to cause heart block. Although her serum lithium level was not measured due to unavailability of the assay at our hospital, her serum paliperidone level was within the therapeutic range (**Table 1**). Based on her medication history and clinical presentation, the combination of lithium and paliperidone was suspected as the likely cause of her hypothyroidism, bradycardia, and sinus arrest.

**Table 1:** Thyroid function tests, serum drug levels, and other laboratory results on presentation.

Test	First evaluation	Second evaluation	Reference Range
**TSH**	9.08 mIU/L	3.60 mIU/L	0.51–4.94 mIU/L
**T4**	45.05 nmol/L	57.92 nmol/L	58.1–140.6 nmol/L
**T3**	1.54 nmol/L	2.06 nmol/L	0.92–2.79 nmol/L
**FT4**	10.94 pmol/L	12.74 pmol/L	11.5–22.7 pmol/L
**FT3**	5.08 pmol/L	6.34 pmol/L	3.5–6.5 pmol/L
**TPOAB**	<28.00 U/ml	<28.00 U/ml	0–60 U/ml
**THGAB**	<15.00 U/ml	<15.00 U/ml	0–60 U/ml
**WBC**	4.88×10^9^/L	5.44×10^9^/L	4.1–11×10^9^/L
**RBC**	4.03×10^12^/L	4.19×10^12^/L	4.1–5.3×10^12^/L
**PLT**	219×10^9^/L	222×10^9^/L	150–407×10^9^/L
**K^+^**	3.66 mmol/L	3.68 mmol/L	3.5–4.9 mmol/L
**Na^+^**	141.00 mmol/L	138.50 mmol/L	137–147 mmol/L
**Cl^-^**	108.10 mmol/L	106.10 mmol/L	98–110 mmol/L
**Ca^2+^**	2.50 mmol/L	2.37 mmol/L	2.1–2.8 mmol/L
**P^5+^**	1.31 mmol/L	1.40 mmol/L	0.93–1.61 mmol/L
**Mg^2+^**	0.87 mmol/L	0.76 mmol/L	0.75–1.02 mmol/L
**LDH**	121 U/L	159 U/L	120–250 U/L
**CK**	43.0 U/L	41.0 U/L	40–200 U/L
**HBDH**	95 U/L	114 U/L	72–182 U/L
**CKMB**	9.00 U/L	10.00 U/L	≤24 U/L
**ALT**	9 U/L	15 U/L	6–29U/L
**AST**	16 U/L	22 U/L	10–30U/L
**UA**	423 μmol/L	308 μmol/L	150–360 μmol/L
**UREA**	2.8 mmol/L	2.8 mmol/L	2.5–6.5 mmol/L
**CREA**	56 μmol/L	46 μmol/L	39–76 μmol/L
**GLU**	4.94 mmol/L	4.94 mmol/L	3.89–6.1mmol/L
**PLPT**	42.71 ng/ml	0.00 ng/ml	20–60 ng/ml

ALT, alanine aminotransferase; AST, aspartate aminotransferase; Ca^2+^, serum calcium concentration; CK, creatine kinase; CKMB, creatine kinase isoenzyme; Cl^-^, serum chlorine concentration; CREA, creatinine; FT3, free triiodothyronine; FT4, free thyroxine; GLU, fasting blood glucose; HBDH, hydroxybutyrate dehydrogenase; K^+^, serum potassium concentration; LDH, lactate dehydrogenase; Mg^2+^, serum magnesium concentration; Na^+^, serum sodium concentration; P^5+^, serum phosphorus concentration; PLPT, paliperidone blood concentration; PLT, platelet count; RBC, red blood cell count; T3, triiodothyronine; T4, thyroxine; TSH, thyroid stimulating hormone; THGAB, anti-thyroglobulin; TPOAB, thyroid peroxidases antibody; WBC, white blood cell count; UA, uric acid; UREA, urea.

This was further supported when her thyroid function normalized 20 days after stopping these medications, without levothyroxine replacement (**Table 1**). Bradycardia and sinus dysfunction were documented to have resolved within the same timeframe as thyroid function stabilization, with a restoration of normal sinus rhythm to 64 beats per minute occurring 20 days after medication cessation. This aligned timing provides evidence that the cardiac issues were likely attributable to drug-induced sinus node effects rather than idiopathic pathology. The half-lives of lithium and paliperidone are 18 hours and 23 hours, respectively, which explains the timing of the resolution of her subclinical hypothyroidism and sinus node dysfunction 20 days after discontinuing these medicines. Then, she was switched to olanzapine 2.5 mg once daily for her psychiatric disorder. Pacemaker implantation was not pursued because her sinus rhythm recovered with drug withdrawal.

## Discussion

To our knowledge, our study first reported that subclinical hypothyroidism and bradycardia without QT prolongation associated with the combination of lithium and paliperidone. Bradycardia can result from various etiologies, including cardiac tissue damage, hypertension, subclinical hypothyroidism, and adverse drug effects. In this case, the onset of bradycardia and subclinical hypothyroidism occurred with medication use, and both resolved after withdrawal of the causative medications. Therefore, it is reasonable to attribute the occurrence of bradycardia and subclinical hypothyroidism to adverse drug reactions. Interestingly, at seven days post-discontinuation, her serum paliperidone level was undetectable (**Table 1**), and lithium had undergone five half-lives of elimination, yet bradycardia persisted. By 20 days after stopping these medications, her thyroid function had normalized, and a 24-hour ECG and 24-hour ambulatory blood pressure monitoring showed a return to normal.

Lithium is a common mood-stabilizing drug for manic and bipolar disorder. Its therapeutic plasma concentration is 0.6 to 1.0 mmol/L. The effects of lithium on thyroid function are well-recognized, but its effects on cardiac function have been less frequently reported. Lithium-induced sinus dysfunction, such as symptomatic bradycardia, and sinoatrial node dysfunction, has been reported even for the recommended therapeutic levels (2, 5, 6). The possible mechanisms underlying lithium’s cardiac toxicity likely involve a dose-dependent sodium channel blockade in myocytes (7). At therapeutic levels, the most common changes of T-wave flattening and sinus node dysfunction are generally benign and asymptomatic. Toxic lithium (>1.5 mmol/L) can cause sinoatrial block, intraventricular conduction delay, Brugada pattern changes, QTc prolongation, ventricular instability, and even sudden cardiac death (1). Lithium-associated bradycardia may also be correlated with drug-induced hypothyroidism. Lithium frequently causes hypothyroidism by impairing multiple aspects of thyroid function, with a prevalence of 37% in women (8). Biochemical findings in a previous study of lithium-induced hypothyroidism showed elevated TSH, decreased FT4, and possibly positive anti-thyroid peroxidase antibody (9), which are consistent with the laboratory results of this patient. Furthermore, lithium is concentrated in the thyroid gland, which interferes with thyroglobulin synthesis and reduces peripheral T4–T3 conversion. In this case, the resolution of bradycardia upon normalization of thyroid status supports the role of lithium-induced hypothyroidism, which is consistent with studies linking hypothyroidism to sinus bradycardia (10).

A previous study demonstrated an association between SGAs (e.g., clozapine, olanzapine, risperidone, etc.) and arrhythmias (11). Moreover, antipsychotics can influence cardiac repolarization and heart rate, potentially resulting in fatal arrhythmias (12). Interestingly, among atypical antipsychotic users, the risk of sudden cardiac death is 2.26 times higher than in non-users, and higher doses are significantly associated with adverse events (13). Additionally, a meta-analysis found antipsychotics confer a greater risk of tachycardia relative to placebos, although no significant differences were seen between antipsychotics and placebos or among antipsychotics for bradycardia (14). However, in this case, the patient developed sinus bradycardia when being treated with the combination of paliperidone and lithium. The discrepancy between our case and prior study findings may have been due to differences in the subjects studied.

Although antipsychotic-induced bradycardia is rare, it can lead to significant consequences. The precise mechanism of paliperidone-regulated heart rate has not been fully elucidated. Most SGAs with mixed receptor activity are associated with bradycardia. Previous studies have demonstrated a link between the 5-hydroxytryptamine (5-HT) receptor and vagal-mediated bradycardia (15–18). For example, bradycardia with amisulpride may relate to an affinity for 5-HT2B and 5-HT7 receptors (19). Similarly, quetiapine-induced bradycardia has been hypothetically attributed to affinities for 5-HT1A and 5-HT2 receptors (20). In this case, paliperidone has affinity for 5-HT2 and 5-HT7 but not 5-HT1A receptors. Therefore, 5-HT2 and 5-HT7 antagonism may underpin paliperidone-associated bradycardia.

The drug–drug interaction between lithium and paliperidone is not well understood. There are two opposing hypotheses regarding interactions between antipsychotics and lithium. One posits that combining antipsychotics with lithium may increase lithium toxicity (21), which could be due to the anticholinergic effects of antipsychotics, including decreased urine output and delayed lithium elimination, resulting in elevated serum lithium levels. Another hypothesis found no significant differences in serum lithium concentrations between groups combining antipsychotics versus single antipsychotic use. Furthermore, a study showed no significant correlation between the lithium dose and serum concentrations (22). Therefore, more studies with larger samples are warranted to explore the interaction between antipsychotics and lithium.

## Limitations

A major limitation of this case report was the lack of serum lithium level measurement due to inadequate equipment at our hospital. Without serum lithium data, we cannot ascertain if the level was within the therapeutic or toxic range. This could affect the interpretation of the causal relationship between lithium and adverse effects. It would be better to monitor the serum lithium concentration and adjust the dose accordingly. Additionally, the lack of baseline cardiological data and serological exams prior to starting medications restricted the ability to fully evaluate the patient’s baseline status and compare changes from the pre-treatment state to during/after pharmacotherapy.

## Conclusion

This case illustrates the potential for lithium and SGA combination therapy to increase the risk of hypothyroidism, bradycardia, and sinus arrest, even at therapeutic doses. Careful monitoring for cardiac effects is advised when using this regimen. Routine ECG and thyroid function testing are recommended, with 24-hour ambulatory ECG monitoring (Holter) as indicated based on concerning symptoms. To mitigate toxicity, clinicians may consider using the lowest effective doses of lithium and antipsychotics when used in combination, along with vigilant monitoring, prompt intervention, judicious prescribing, and patient/caregiver education. Further research on the cardiac and endocrine effects of lithium-antipsychotic polypharmacy can help refine prescribing practices and monitoring protocols to optimize patient safety. In summary, this case underscores the importance of vigilance and individualized management when combining mood stabilizers and antipsychotics to balance risks and benefits.

## Data availability statement

The original contributions presented in the study are included in the article/supplementary material. Further inquiries can be directed to the corresponding authors.

## Ethics statement

This study was approved by the Ethics Committee of the Second People’s Hospital of Honghe Hani and Yi Autonomous Prefecture. All procedures performed in studies involving human participants were in accordance with the ethical standards of the institutional and/or national research committee and with the 1964 Helsinki declaration and its later amendments or comparable ethical standards. The studies were conducted in accordance with the local legislation and institutional requirements. Written informed consent was obtained from the minor’s legal guardian for the publication of any potentially identifiable images or data included in this article.

## Author contributions

WW: Formal analysis, Investigation, Methodology, Visualization, Writing – original draft. YY: Investigation, Methodology, Resources, Writing – original draft. HW: Conceptualization, Project administration, Supervision, Writing – original draft. YY: Conceptualization, Supervision, Writing – original draft. JL: Methodology, Project administration, Writing – review & editing. JL: Funding acquisition, Investigation, Writing – original draft.

## References

[1] Mehta N Vannozzi R. Lithium-induced electrocardiographic changes: A complete review. Clin Cardiol (2017) 40:1363–7. doi: 10.1002/clc.22822 PMC6490621 29247520

[2] Ataallah B Al-Zakhari R Sharma A Tofano M Haggerty G. A rare but reversible cause of lithium-induced bradycardia. Cureus (2020) 12:e8600. doi: 10.7759/cureus.8600 32676239 PMC7362593

[3] Jarema M Bieńkowski P Heitzman J Parnowski T Rybakowski J. Paliperidone palmitate: effectiveness, safety, and the use for treatment of schizophrenia. Psychiatr Pol (2017) 51:7–21. doi: 10.12740/pp/64581 28455891

[4] Sharma N Bhat S Ravi D Ochieng P. Severe hypothermia, bradycardia and cardiac arrest in association with risperidone. BMJ Case Rep (2020) 13:e234999. doi: 10.1136/bcr-2020-234999 PMC7247412 32439747

[5] Goldberger ZD. Sinoatrial block in lithium toxicity. Am J Psychiatry (2007) 164:831–2. doi: 10.1176/ajp.2007.164.5.831 17475748

[6] Sarangi A Javed S Paul T Amor W. Lithium-induced sinoatrial node dysfunction. Cureus (2021) 13:e16778. doi: 10.7759/cureus.16778 34513385 PMC8404648

[7] Oudit GY Korley V Backx PH Dorian P. Lithium-induced sinus node disease at therapeutic concentrations: linking lithium-induced blockade of sodium channels to impaired pacemaker activity. Can J Cardiol (2007) 23:229–32. doi: 10.1016/s0828-282x(07)70750-x PMC2647873 17347696

[8] Henry C. Lithium side-effects and predictors of hypothyroidism in patients with bipolar disorder: sex differences. J Psychiatry Neurosci (2002) 27:104–7. PMC161639 11944505

[9] Livingstone C Rampes H. Lithium: a review of its metabolic adverse effects. J Psychopharmacol (2006) 20:347–55. doi: 10.1177/0269881105057515 16174674

[10] Nakanomori A Nagano N Seimiya A Okahashi A Morioka I. Fetal sinus bradycardia is associated with congenital hypothyroidism: An infant with ectopic thyroid tissue. Tohoku J Exp Med (2019) 248:307–11. doi: 10.1620/tjem.248.307 31462599

[11] Pacher P Kecskemeti V. Cardiovascular side effects of new antidepressants and antipsychotics: new drugs, old concerns? Curr Pharm Des (2004) 10:2463–75. doi: 10.2174/1381612043383872 PMC2493295 15320756

[12] Stoner SC. Management of serious cardiac adverse effects of antipsychotic medications. Ment Health Clin (2017) 7:246–54. doi: 10.9740/mhc.2017.11.246 PMC6007733 29955530

[13] Ray WA Chung CP Murray KT Hall K Stein CM. Atypical antipsychotic drugs and the risk of sudden cardiac death. N Engl J Med (2009) 360:225–35. doi: 10.1056/NEJMoa0806994 PMC2713724 19144938

[14] Huhn M Arndt T Schneider-Thoma J Leucht S. Effects of antipsychotics on heart rate in treatment of schizophrenia: a systematic review and meta-analysis. Ther Adv Psychopharmacol (2022) 12:20451253221097261. doi: 10.1177/20451253221097261 35774251 PMC9237927

[15] Bogle RG Pires JG Ramage AG. Evidence that central 5-HT1A-receptors play a role in the von Bezold-Jarisch reflex in the rat. Br J Pharmacol (1990) 100:757–60. doi: 10.1111/j.1476-5381.1990.tb14088.x PMC1917580 2207497

[16] Pires JG Silva SR Ramage AG Futuro-Neto HA. Evidence that 5-HT3 receptors in the nucleus tractus solitarius and other brainstem areas modulate the vagal bradycardia evoked by activation of the von Bezold-Jarisch reflex in the anesthetized rat. Brain Res (1998) 791:229–34. doi: 10.1016/s0006-8993(98)00109-7 9593908

[17] Damaso EL Bonagamba LG Kellett DO Jordan D Ramage AG MaChado BH. Involvement of central 5-HT7 receptors in modulation of cardiovascular reflexes in awake rats. Brain Res (2007) 1144:82–90. doi: 10.1016/j.brainres.2007.01.088 17320834

[18] Ramage AG Villalón CM. 5-hydroxytryptamine and cardiovascular regulation. Trends Pharmacol Sci (2008) 29:472–81. doi: 10.1016/j.tips.2008.06.009 19086344

[19] Huang LC Huang LY Tseng SY. Amisulpride and symptomatic bradycardia: a case report. Gen Hosp Psychiatry (2015) 37:e491–492. doi: 10.1016/j.genhosppsych.2013.12.005 26162544

[20] Nakamura M Seki M Sato Y Nagamine T. Quetiapine-induced bradycardia and hypotension in the elderly-a case report. Innov Clin Neurosci (2016) 13:34–6. PMC4896827 27413585

[21] Netto I Phutane VH Ravindran B. Lithium neurotoxicity due to second-generation antipsychotics combined with lithium: A systematic review. Prim Care Companion CNS Disord (2019) 21:17r02225. doi: 10.4088/PCC.17r02225 31237432

[22] Obara R Tomita T Goto H Kohda Y Yoshida T Kudo K. Effect of antipsychotics on serum lithium levels and white blood cell counts. Neuropsychopharmacol Rep (2021) 41:532–7. doi: 10.1002/npr2.12210 PMC8698700 34687178

